# Usage of power by different types of trainers in the education of paramedics – evaluation by means of a validated questionnaire

**DOI:** 10.3205/zma001501

**Published:** 2021-09-15

**Authors:** Melanie Misamer, Markus Flentje, Alexander Stötefalke, Hendrik Eismann

**Affiliations:** 1 Hochschule für Angewandte Wissenschaft und Kunst, Göttingen, Germany; 2 Hannover Medical School, Department of Anaesthesiology and Intensive Care Medicine, Hannover, Germany; 3 Johanniter-Akademie Bildungsinstitut Niedersachsen/Bremen, Hannover, Germany

**Keywords:** emergency medical service, dimension of power, practical instructor, medical educator, high risk organization

## Abstract

**Objective: **Emergency medical services are characterized by a high pressure to act. Dealing with trainees is a challenge. It is known, that the use of power in education subsists: power can be applied in a participative and restrictive way. We investigated the transferability of existing scales to the education system of Emergency medical service trainees. We hypothesized: a restrictive (a) and participative (b) use of power, can be demonstrated in Emergency medical service training, (c) the use of power by educators, who are responsible for theoretical learning, and instructors, who accompany trainees in real-life emergencies, are different and (d) the assessed participatory and restrictive use of power by trainers is negatively correlated.

**Methods: **In a cross-sectional study, 206 trainees of Emergency medical service schools completed a questionnaire. The survey consists of 35 power related items regarding medical educators and practical instructors. Differences in the dimensions of power application were tested. The effect size and the correlation between power dimension were calculated.

**Results: **The reliability of the scales was .92 (practical instructor) and .89 (medical educator) by removing one item. All subscales showed values with higher Cronbach’s alpha than .68. Application of participative power differs (p<.00) between practical instructors (mean 64.7; SD 20.3) and medical educators (mean 55.3; SD 17.8). The participatory and the restrictive use of power correlated for medical educators significant negatively (r=-.48; p<.01).

**Conclusion:** In both educator and instructor groups the use of participative power had a greater agreement that the use of restrictive techniques. The practical instructors used participative power slightly more often that did educators due to the dependency on the trainee as a team member. The context of the scales partially overlaps with other descriptions such as leadership and instructor quality.

## Zusammenfassung

**Hintergrund:** Die Tätigkeit im Rettungsdienst ist durch einen hohen Handlungsdruck gekennzeichnet. Der Umgang mit Auszubildenden ist eine Herausforderung. Es ist bekannt, dass in der Ausbildung Macht eingesetzt wird: Macht kann partizipativ und restriktiv eingesetzt werden. Wir haben in dieser Arbeit die Übertragbarkeit bestehender Skalen auf das Ausbildungssystem von Auszubildenden im Rettungsdienst untersucht. Wir haben folgende Hypothesen aufgestellt: ein restriktiver (a) und partizipativer (b) Machtgebrauch kann in der Notfallsanitäterausbildung nachgewiesen werden, (c) der Machtgebrauch von medizinischen Lehrkräften, die für das theoretische Lernen verantwortlich sind, und Praxisanleitern ist unterschiedlich und (d) der eingeschätzte partizipative und restriktive Machtgebrauch von Ausbildern korreliert negativ.

**Methoden: **In einer Querschnittsstudie nahmen 206 Auszubildende in Notfallsanitäterschulen an der Fragebogen-Erhebung teil. Der Fragebogen beinhaltet jeweils 35 machtbezogene Items in Bezug zu medizinischen Lehrkräften und Praxisanleitern. Es wurden Unterschiede in den Dimensionen der Machtanwendung überprüft. Die Effektgröße und die Korrelation zwischen den Machtdimensionen wurden berechnet.

**Ergebnisse: **Die Reliabilität der Skalen lag – nachdem ein Item entfernt wurde – bei 0,92 (Praxisanleiter) und 0,89 (medizinische Lehrkräfte). In allen Subskalen lagen das Cronbachs Alpha höher als 0,68. Die Anwendung der partizipativen Macht unterscheidet sich (p<0,01) zwischen Praxisanleitern (Mittelwert 64,7; SD 20,3) und medizinischen Lehrkräften (Mittelwert 55,3; SD 17,8). Der partizipative und der restriktive Machtgebrauch korrelierten für medizinische Lehrkräfte signifikant negativ (r=-0,48; p<0,01).

**Schlussfolgerungen:** In den beiden Gruppen – medizinische Lehrkräfte und Praxisanleiter – war die Bewertung des partizipativen Machtgebrauchs größer als des restriktiven. Die Praxisanleiter setzten partizipative Macht etwas häufiger ein als die Lehrkräfte, was auf die Abhängigkeit vom Auszubildenden als Teammitglied zurückzuführen ist. Der Kontext der Skalen überschneidet sich teilweise mit anderen Beschreibungen wie Führung und Ausbilderqualität.

## Introduction

Emergency medical services (EMS) operate under the conditions of a high responsibility team (HRT). The work environment is characterized by complex and demanding work contexts. Errors lead to severe consequences like patient harm [1]. Success factors for avoiding accidents in HRT are non-technical skills, like task management, decision making, situational awareness and teamwork [2].

Power is a foundation for leadership and teamwork. Power application has a direct influence on incident management and is even more if a trainee is involved. Schmalt and Heckhausen summarized power definitions in the following way: “when someone is able to cause another to do something, he would not do otherwise” [3].

The relationship between trainee and trainer is clearly characterized by differences in hierarchy. The use of power could have an influence on the interacting-promoting basic elements “experience of justice and trust” ([4] p.1). Therefore the application of power has a direct influence on learning effects [5].

Using power in a teacher-trainee relationship against the interest of the trainee and the learning process can be defined as restrictive. Examples of published restrictive power applications are bad-mouthing, blaming, physical or sexual violation [6]. Negative relations with teachers are perceived as very stressful [7]. Participative power application take place when used in the interest of the trainee and the whole process (in terms of a “win-win”-situation). Dorst understands this power as means to empower, support and promote others [8].”Showing appreciation” and “admitting one’s own mistakes” are general important principles of this power application [7]. A teacher can use power to praise trainees, encourage them to perform better, and motivate them.

German EMS is an emergency physician-based system. Ambulances are manned with a paramedic and a co-worker (EMT with six-month training or a paramedic-in-training – second or third year of three) and meet with the emergency physician on scene.

Paramedic and co-worker regularly have to bridge time until the arrival of the emergency physician by using invasive medical procedures. Consequently, the training objectives of the trainees are to carrying out invasive procedures (e.g. endotracheal intubation and ventilation) to avert hazard – or providing care for patients in standardized emergency situations (e.g. drug application in hypertensive crises) without the presence of an emergency physician [https://www.gesetze-im-internet.de/notsang/BJNR134810013.html]. Most of the practical education of a paramedic takes place in a real emergency situation and is supervised by a paramedic specialized in on-the-job training (practical instructor). Theoretical training takes place in rescue schools. There, medical educators train during lectures, practical exercises and simulation cases. These trainers have a paramedic degree but are often no longer actively involved in EMS.

The aim of the study was to evaluate the transferability of the use of power from school to a rescue service organization. The results can be a basis for interventions to improve incident management and increase training effects. The mechanisms of power should be trans-sectoral [9]. Our hypotheses were:

1. a restrictive use of power, as demonstrated in other educational settings, can also be demonstrated in rescue service training,
2. a participatory use of power, as demonstrated in other educational settings, can also be demonstrated in rescue service training,
3. the use of restrictive and participative power by medical educators and practical instructors are different, and
4. that the assessed participatory and restrictive use of power by trainers is negatively correlated.

## Materials and methods

### Research design

The study is a cross-sectional study with participants from two EMS schools in the province of lower saxony in Germany. All participants were in training for paramedic. The authors (MF or HE) visited the participants in school and explained the aims and the process of the study. A QR (Quick Response) code with a link to online survey was presented to the participants so they were able to conduct the survey via a tablet computer or a smartphone. The survey consists of two questionnaires – one with reference to the teachers of the rescue school (medical educator) and one with regard to the practical instructors. Participation was voluntary for all trainees. According to the specifications of the ethics committee, the survey was anonymized. Data was gathered in a three-month period. This study was reviewed and approved by the ethics committee of the Hannover Medical School (No. 7858_BO_K_2018).

#### Measures

The entire questionnaire contained 70 power related items (35 for medical educators and 35 identical items for practical instructors. Sex and the actual year of training was registered as demographic data. The questionnaire “MVU_S_rel” (“Macht, Vertrauen und Ungerechtigkeit für Schüler*innen aus relationaler Perspektive”) was initially developed for schools (grade five to ten) and has satisfactory reliabilities in this environment [4]. This measurement instrument is a factor-analytical symbiosis of measurement instruments from psychological leadership research [10] and school research [11]. The advantage of this questionnaire is, that power can be analyzed more differentiated (participative and restrictive). Usage of participative power was represented by the three scales “trust” (11 Items), “supportive care” (7 Items) and to “collaborative opportunities” (3 Items). The use of restrictive power was represented by the five scales “restrictive consequences” (6 items), “shaming” (2 items), “lack of egality” (2 items), “injustice” (2 items) and “duress” (2 Items). The entire questionnaire is shown in the appendix. The questionnaire is validated in German language and in the German culture sphere. Internal consistency of the subscales (teacher/scholar) “trust” (α=.88/α=.91), “supportive care” (α=.92/α=.91), “involvement” (α=.63/α=.79) and “lack of egality” (α.=.87/α=.72), “injustice” (α=.79/α=.81), “blaming” (α=.89/α=.82), “force” (α=.88/α= 73) “restrictive consequences” (α=.86/α=.81) proved to be satisfactory. The representation of the English version in the appendix is for the readers’ understanding.

The questionnaire was adapted in two aspects: the participants had the opportunity to rate the items on a unipolar scale between from 0 to 100 using a slider. This allows a higher validity and reliability of a test [12], [13]. The participants could not see the deposited numbers and moved the slider between “does not apply” (=0) and “strongly applies” (=100). The wording of the items was adapted in the context of medical education – instead of the “teacher”, the terms “practical instructor” and “medical educator” were used.

#### Data analysis

Demographic survey data were analyzed in a descriptive manner. The reliability of the scales was determined by Cronbach’s alpha. Since the scales’ reliability were satisfactory, no factor analysis was performed. Instead, an 2^nd^ order factor analysis was conducted and the assignment of the items to the participative and restrictive power-dimensions were checked (hypothesis (a) and (b)). The condition for the feasibility of the factor analysis was verified by the Kaiser-Meyer-Olkin test (KMO) and Bartlett test. In order to test hypothesis (c) a t-test for dependent samples was conducted after testing for normal distribution by Kolmogorov-Smirnov test. For the effect size Cohens d was evaluated. Pearson’s correlation coefficient (PCC) is used to measure the correlation between dimensions of power application to test hypothesis (d). For all statistics, SPSS 24 (IBM Corporation, USA) was used.

## Results

Overall, the survey was completed by 216 participants. Due to incomplete data, 206 questionnaires were included into the analysis. Sixty-nine (33.5%) participants were female, 137 (66.5%) were male. The age varied between 18 and 36 years (M=23.06; SD=3.72). The state of training ranged from first year (41.3%) to second (26.2%) and third year (32.5%). The participants had training contracts (for their practical training) with non-government-organizations (83%), local government organizations (7.8%) and fire departments (2%).

### Reliability of the scales

In order to test hypothesis (a) and (b) Cronbach’s alpha was calculated. By removing the item “trainees are allowed to have a say in how rooms are designed”, Cronbach’s alpha could be increased for practical instructor and for medical educator. All subscales resulted in a good to very good reliability. Detailed data is shown in table 1 (Tab. 1).

The 2^nd^ order factor analyses were performed to verify the mapping of the subscales to the dimensions of power in the EMS culture sphere. The quality markers comply with the requirements. Practical instructors: KMO: .85; Barlett: .00; Medical educator: KMO .81, Barlett .00. Assuming a cut off value of .3, all items fit the proposed dimension of power. All data is shown in table 2 (Tab. 2) and table 3 (Tab. 3).

#### Difference in power application by practical instructors and medical educators

**Figure 1:**
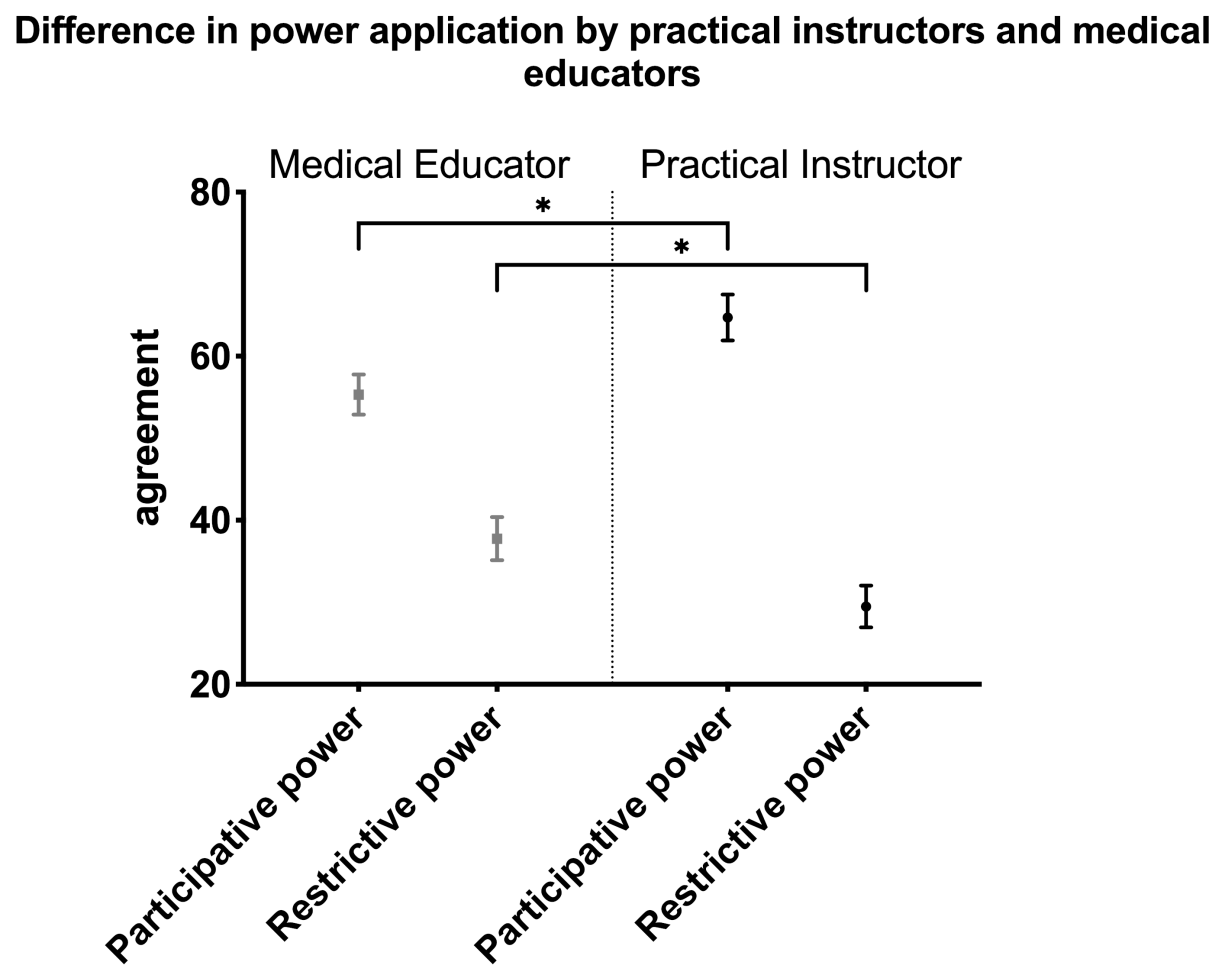
Measured difference in power application (n=205). Classification of agreement: 1= disagree strongly to 100= agree strongly; * = significant p< .00. Cohens d participative power (.50) and restrictive power (.43); medical educator: participative power: mean 55.3, SD 17.8, restrictive power: mean 37.8, SD 19.1, t(204) -5.09; practical instructor: participative power: mean 64.7, SD 20.34, restrictive power: mean 29.5, SD 18.5, t(204) 5.7; range:0-100. SD: standard deviation.

Hypothesis (c) was tested using a t-test for dependent samples. Normal distribution was given for all data (Kolmogorov-Smirnov test). Application of participative power is 64.7 (SD 20.3) for practical instructors and 55.3 (SD 17.8) for medical educators, respectively (range: 0-100). Application of restrictive power is 29.5 (SD 18.5) for practical instructors and 37.8 (SD 19.1) for medical educators. Both differences are significant (p<.00). Data is shown in figure 1 (Fig. 1).

#### Correlation 

The correlation of the participatory and restrictive dimension of the application of power was determined by Pearson’s correlation coefficient. The participatory and the restrictive use of power correlated for medical educators significant negatively (r=-.48; p<.01). The effect size is medium [14]. The correlation among the dimensions of the practical instructors was also significant negatively (r=-.64; p<0.01). The correlation is large table 4 (Tab. 4).

## Discussion

### Application of the scales

Our primary hypothesis of the study was to test the existing scales for measuring power [4] in the environment of an emergency medical service in a context of theoretical (medical educator) and practical (practical instructor) training. Our results show a good reliability of the scales. The items could be mapped with the dimension of power as proposed in the school-based questionnaire. The relationship between trainer and trainee therefore seems to follow interpersonal relationships regardless of the environment. In an earlier study group, we developed a quality management tool with competences of practical instructors [14]. Items like “Our practical instructors neither argue with nor humiliate us in front of the patient” and “Our practical instructors see us as fully-fledged team members and involve us” fit to the scales “shaming” and “collaborative opportunities” of the power questionnaire [15]. Thurgur et. al. investigated the specific situation of training in the emergency environment [16] and found comparative items like “positive teacher attitude”, “treats residents as a colleague (respect)”. So, the participative use of power seems to be a core competence for being a good trainer in a healthcare environment.

Power in healthcare is usually used in the context of management [17]. We often interpret differences in application of power – such as rank and know-how to be the basis for leadership and teamwork. The application of power is comparable to the item “using authority and assertiveness” of the Anesthetists’ Non-Technical Skills (ANTS) rating framework [18]. Training of non-technical skills in so called crisis resource management courses have a direct impact on patient outcome [19] and consumption of funds [20]. The application of participative or restrictive power has subsequent effects on Teamwork. Detailed consideration of power can offer chances to further investigate the context of teamwork.

To our knowledge, there are no research results, which correlate the use of power with learning effects of trainees in HRTs. The trainees in our target groups are in different age and life situations compared to the school environment. A total analogy to the school sector is not given due to the different age and life situation of the trainees. Nevertheless, a hypothesis may be formulated, because it is known that behavior of teachers influences learning [21]. Subgroup analyses on the part of the trainees would be interesting to analyze possible influences such as school history, culture, age and previous professional education. The fields of competency proposed by Srinivasan et al. also include terms as “social and communicative competences” and “role model and professional behavior” [22] and overlap in content with scales of our power questionnaire. Objective criteria of learning success would have to be worked out, since passing of the final exam in over 90% is very high [23].

#### Differences in power use

Our findings show, that the application of participative power of practical instructors is significantly higher than by medical educators. Although we asked for groups of people, the teaching and learning environment between classroom and emergency ambulance differ in location and situation. In fact, the school environment offers unexcited situations due to the missing pressure of real patient care. However, theoretical instructions are also characterized by a difference in the number of trainees and medical educators. Therefore, there will rarely be situations with one educator and one trainee. Building of personal relationships is more difficult. The method of simulation is widely used in rescue service schools for learning patient care procedures. A team of trainees have to perform while other trainees and the educators observe the scenario. This situation and a debriefing as its core element [24] involve the risk of blaming [25]. The aspect blaming is one component of our used questionnaire. The more frequent use of simulation-based training in rescue service schools could explain why the medical educator were rated to use restrictive power more often. Increasing competencies in debriefing techniques could possibly improve the use of power. As far as we know, there are no studies on correlation of the use of power and the method simulation. The work environment of an EMS station offers much more potential. In standby time between rescue missions, teams might spend time together. Everyday activities like cooking and watching television are carried out together.

The practical instructor is more dependent on the learning success and emergency competencies of the trainee. He has to compensate a possible gap in performance of the trainee and has higher interests in practical training. However, this does not yet conclude the positive supportive use of power.

The practical instructors are well-trained, internalized the importance of non-technical skills and know about the conditions and importance of “speaking up”. “Speaking up” is defined as intentionally expressing work-related ideas, information and options and is a success factor in critical patient care [26]. Strong hierarchies prevent a successful speaking up [27]. According to our interpretation, the closer personal contact, the mutual dependency and the knowledge of non-technical skills lead to a more participatory use of power by the practical instructors.

#### Correlation

We found a negative correlation of participative and restrictive power for both practical instructors and medical educators. This can be seen as an indication that trainers do rather not vary their behavior, as other researchers found [28], [29]. But we do not consider the measurement depending on the situation. Emergency medicine is characterized by a high variability of time and action pressure. This effect could have more influence on the practical instructors due to their education job in real emergency situations. Further research with a focus on these relationships would be interesting. Another point of research could be whether the use of power can be changed through training ([4] p.1). Here the questionnaire could be used as an evaluation tool.

#### Limitations

Emergency services in Germany are organized very heterogeneous due to the non-central administration by the individual federal states. The conditions of invasive procedures to critical patients are given by a local medical director. This heterogeneity may influence the transferability of our work to another EMS. We tried to reduce this effect by including two rescue schools which recruit their trainees over the complete federal state of Lower Saxony. Even if the scales – developed in a school culture sphere – allow a good applicability for the EMS training, it may not be the complete power spectrum of this area. There might be EMS-specific items, which we could not yet present with our methodology. The development of EMS-specific items requires a redevelopment of items e.g., by qualitative research interviews with explorative factor analyses. It is questionable whether there are healthcare-specific item or areas like e.g., emergency room, EMS, operation theatres and delivery rooms had to be considered individually.

We understand our study as a start in research on power application in high responsibility organizations, so that we see our approach in line with the aims of the study.

## Conclusions

By using a validation process, we were able to transfer the questionnaire in its applicability to the training situation in a rescue service. This questionnaire represents a novelty for evaluation of German-speaking countries. The practical instructors use participative techniques slightly more often that do educators. We interpreted reasons such as closer personal contact, mutual dependence and influence on one´s own work performance. The conceptuality of the power questionnaire overlaps with terms from the field of non-technical- skills with teamwork and leadership. Further research in power application of the rescue service education offers the possibilities for better understanding the context of non-technical skills in an educational environment and learning performance of trainees.

## Acknowledgements

We gratefully thank Mrs. Deniz Böhmelt for the support at the acquisition of data at the German Red Cross Training Center in Hannover, Germany.

## Declarations

### Funding

The study was funded exclusively by the department.

#### Ethics approval

The study was reviewed and approved by the Ethics Committee of the Hannover Medical School (No. 7858_BO_K_2018).

#### Availability of data and materials

The datasets used and/or analyses during the current study are available from the corresponding author on reasonable request.

#### Authors' contributions

The authors MM and MF contributed equally to this work.

All authors listed have contributed sufficiently to the project (MM: conception and design of the study, acquisition of data, analysis and interpretation of data, drafting the manuscript; MF: conception and design of the study, acquisition of data, analysis and interpretation of data, drafting the manuscript; AS: conception of the study, drafting the manuscript; HE: conception and design of the study, analysis and interpretation of data, drafting the manuscript). Authorships have been acknowledged appropriately.

## Competing interests

The authors declare that they have no competing interests. 

## Figures and Tables

**Table 1:**
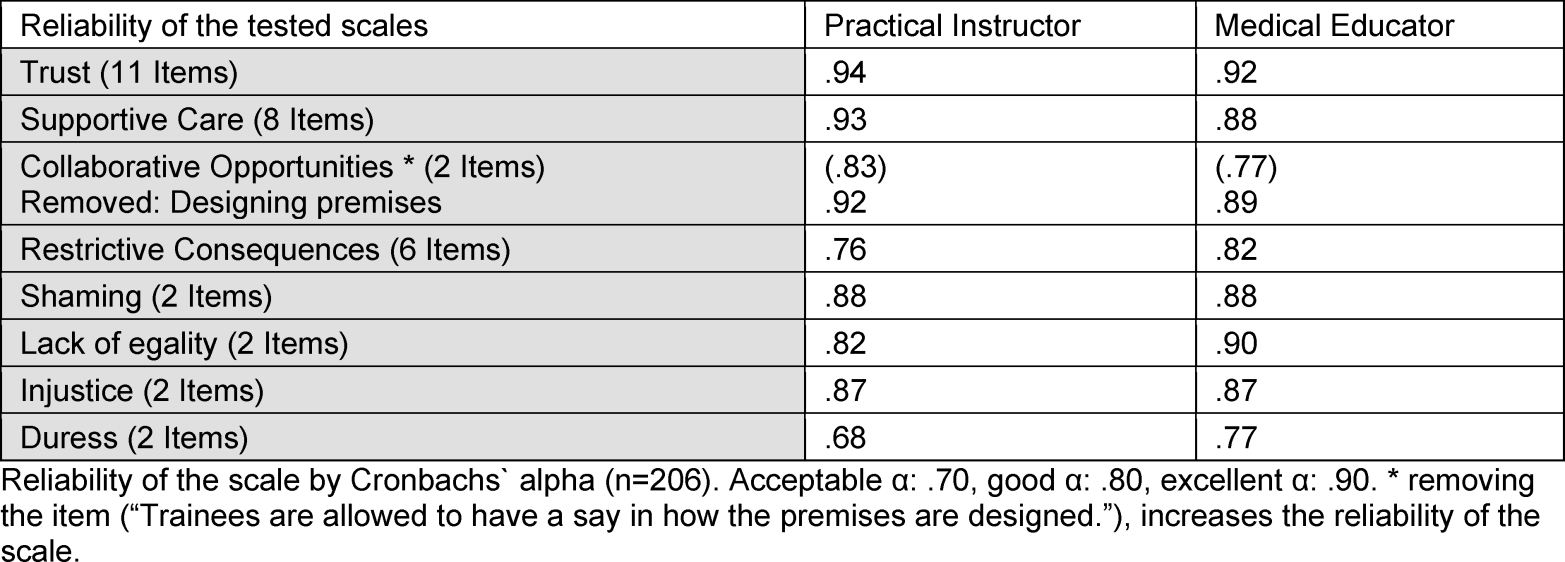
Reliability of the tested scales

**Table 2:**
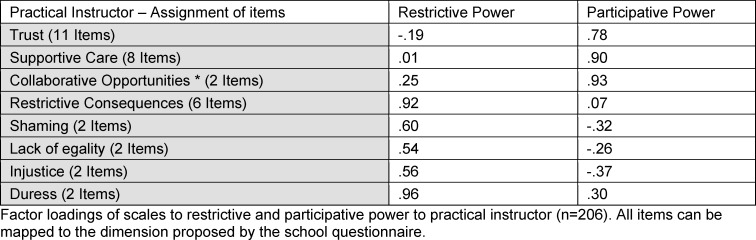
Practical instructor – assignment of items

**Table 3:**
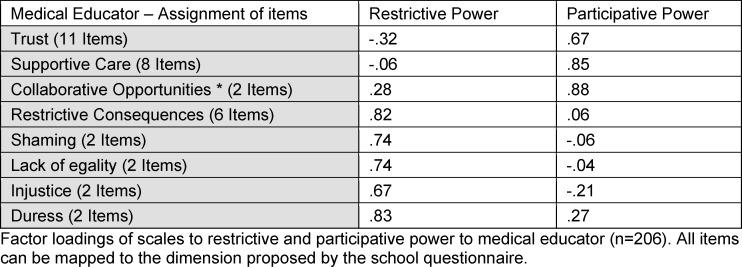
Medical educator – assignment of items

**Table 4:**
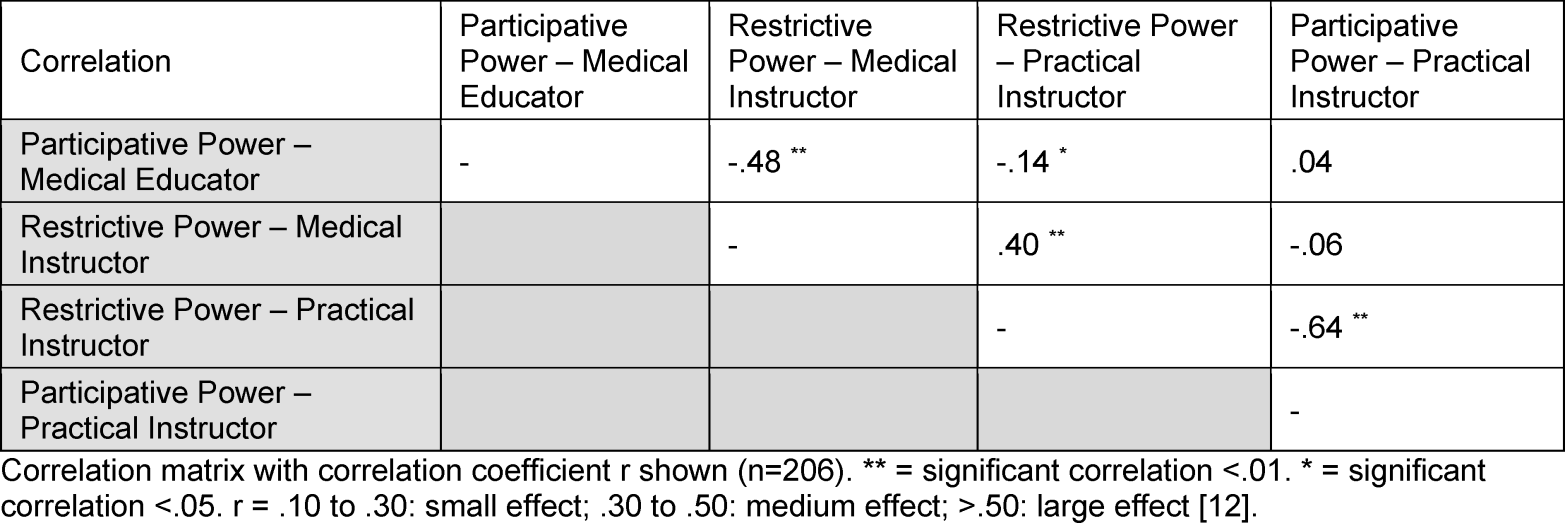
Correlation
